# Editorial: Women in science: allergy research

**DOI:** 10.3389/falgy.2025.1601916

**Published:** 2025-04-24

**Authors:** Gemma Carreras-Badosa, Vanesa Esteban, Vivian Hernandez-Trujillo, Elina Toskala, Manali Mukherjee, Saba Al Heialy, Karin Fieten

**Affiliations:** ^1^ Department of Biology, Faculty of Sciences, University of Girona, Girona, Spain; ^2^ Department of Allergy and Immunology, IIS-Fundación Jiménez Díaz, UAM, Madrid, Spain; ^3^ Herbert Wertheim College of Medicine, Florida International University, Miami, FA, United States; ^4^ Department of Otolaryngology–Head and Neck Surgery, Thomas Jefferson University, Philadelphia, PA, United States; ^5^ Division of Respirology, Department of Medicine, Faculty of Health Sciences, McMaster University, Hamilton, ON, Canada; ^6^ Department of Basic Medical Sciences, College of Medicine, Mohammed bin Rashid University of Medicine and Health Sciences, Dubai, United Arab Emirates; ^7^ Swiss Institute of Allergy and Asthma Research (SIAF), University of Zurich, Davos, Switzerland; ^8^ Dutch Asthma Center Davos, Davos, Switzerland

**Keywords:** allergen, biomarker, anaphylaxis, RNA, drug hypersensitivity, women, allergy

**Editorial on the Research Topic**
Women in science: allergy research

The Allergy research field has seen remarkable progress in recent decades. From elucidating the immunological mechanisms underlying allergic reactions to developing targeted therapies, advancements in this field have had a profound impact on public health worldwide. Amidst these achievements, the contributions of female scientists have often gone underrecognized. This editorial seeks to highlight the essential role women have played—and are still playing—in Allergy research.

Historically, female scientists have faced numerous societal and cultural barriers, including restricted access to advanced education, gender biases in hiring and funding decisions, lower visibility in professional networks, insufficient representation in leadership positions, and limited possibilities to combine care duties with a career. Despite these obstacles, many women have been at the forefront of transformative discoveries in immunology and allergy research. Their perseverance and innovative thinking have led to breakthroughs that not only deepen our understanding of allergies but also drive the development of safer, more effective treatments for patients worldwide.

In this research topic, 5 papers are selected that showcase important advances in the allergy research field.

The mini-review by Zubeldia-Varela et al., titled “*Allergy-associated biomarkers in early life identified by Omics technique*”, emphasizes the importance of identifying predictive biomarkers for allergic diseases, such as food allergy and atopic dermatitis, during the early stages of life. Understanding these early biomarkers is essential for comprehending immature immune responses and developing effective therapeutic approaches. The review highlights three major biomarker categories: the proteome, microbiome, and metabolome, and it also states that the microbiome and its metabolites can influence and modify host responses. The authors stress that elucidating the mechanisms underlying allergic diseases and the integration of omics data is crucial for advancing toward predictive, preventive, and personalized medical strategies.

The systematic review and meta-analysis by Pühringer et al., titled “*Population-based incidence of all-cause anaphylaxis and its development over time: a systematic review and meta-analysis*”, sought to determine the incidence of all-cause anaphylaxis worldwide. Overall, 46 cases of anaphylaxis out of 100,000 population per year were described. The authors found a yearly increase of anaphylaxis incidence of 7.4%, and children were less likely to have anaphylaxis than the general population. This review provides information about this important topic, and further studies could evaluate the reasons for increasing incidence of anaphylaxis.

In an original article published by Fernández-Bravo et al., titled “*Circulating serum profile of small non-coding RNAs in patients with anaphylaxis beyond microRNAs*”*,* differential and specific profiles of small non coding RNAs (sncRNAs) in children with food-mediated anaphylaxis and in adults with drug-mediated anaphylaxis are described. Among the different snoRNAs, snRNAs, tRFs and YRFs, only three molecules (Y_RNA.394, Y_RNA.781 and SCARNA2) were common to both analyses. Beyond miRNAs, it is the first time that snRNAs have been postulated as biomarkers of anaphylaxis. Although preliminary data, studies like this define molecular clues into mechanisms underlying anaphylaxis that would lead to new clinical strategies.

An original research article by Kühl et al., titled “*Cofactors of Drug Hypersensitivity – a mono-center restrospective analysis*”, analyzes 393 patients with suspected drug hypersensitivity reactions (DHR), focusing on drug-specific cofactors influencing confirmed hypersensitivities. Drug provocation tests and statistical modeling identified key associations: obesity was linked to antibiotic hypersensitivity, while atopic dermatitis, elevated IgE, and hypertension correlated with hypersensitivity to non-opioid analgesics. Negative associations for antibiotic hypersensitivity included female sex, allergic rhinitis, hypertension, and elevated IgE. These findings emphasize that certain comorbidities act as cofactors for DHR and could improve risk assessment for drug provocation testing, supporting more targeted and safer allergy diagnostics in clinical practice.

The original article by Reinmuth-Selzle et al., titled “*Chemical modification by peroxynitrite enhances TLR4 activation of the grass pollen allergen Phlp5*”, investigates the chemical modifications of aeroallergens that may contribute to the high prevalence of respiratory allergies in industrialized countries. They discovered that the physiological oxidant peroxynitrite induces chemical modifications and enhances the activation of Toll-like receptor 4 (TLR4) by the grass pollen allergen Phlp5. This finding suggests that direct activation of TLR4 by Phlp5 may play a significant role in sensitization to this major airborne allergen, particularly during oxidative stress. Gaining a deeper understanding of the chemical modifications of allergens and their relationship to immune responses will help develop new immunotherapy treatments.

Today, as global rates of allergies continue to rise, there is a growing need for fresh perspectives and interdisciplinary collaboration to develop inclusive, patient-centered care. Promoting the careers of women in science is critical to meeting these urgent demands. By empowering female researchers, we can catalyze novel strategies for diagnosing, managing, and ultimately preventing allergic diseases. However, increasing women's representation in science goes beyond merely filling positions in labs or research institutions. It necessitates systemic changes, including funding structures that support women-led projects, mentorship programs that guide junior researchers, and policies that encourage and enable a healthy work-life balance.

Showcasing the stories of notable women in allergy research, like in this research topic, can inspire the next generation of women to pursue research careers in allergy, knowing that opportunities for growth and leadership exist. Women in allergy research have already made significant strides in advancing our understanding of allergies. By championing the successes of female researchers in this critical field, we can ensure that the future of allergy research is as innovative, robust, and impactful as possible.

